# Unusual multisystemic involvement and a novel *BAG3* mutation revealed by NGS screening in a large cohort of myofibrillar myopathies

**DOI:** 10.1186/s13023-014-0121-9

**Published:** 2014-08-01

**Authors:** Anna-Lena Semmler, Sabrina Sacconi, J Elisa Bach, Claus Liebe, Jan Bürmann, Rudolf A Kley, Andreas Ferbert, Roland Anderheiden, Peter Van den Bergh, Jean-Jacques Martin, Peter De Jonghe, Eva Neuen-Jacob, Oliver Müller, Marcus Deschauer, Markus Bergmann, J Michael Schröder, Matthias Vorgerd, Jörg B Schulz, Joachim Weis, Wolfram Kress, Kristl G Claeys

**Affiliations:** 1 Department of Neurology, RWTH Aachen University, Aachen, Germany; 2 Institute of Neuropathology, RWTH Aachen University, Aachen, Germany; 3 Centre de Référence des Maladies Neuromusculaires, Nice Hospital and UMR CNRS6543, Nice University, Nice, France; 4 Department of Human Genetics, University of Würzburg, Würzburg, Germany; 5 Department of Neurology, Saarland University, Homburg/Saar, Germany; 6 Department of Neurology, Neuromuscular Center Ruhrgebiet, University Hospital Bergmannsheil, Ruhr-University Bochum, Bochum, Germany; 7 Department of Neurology, Klinikum Kassel, Kassel, Germany; 8 Department of Neurology, Klinikum Merzig, Merzig, Germany; 9 Department of Neurology, Neuromuscular Reference Center, University Hospital Saint-Luc, Brussel, Belgium; 10 Institute Born-Bunge, University of Antwerpen, Antwerpen, Belgium; 11 Neurogenetics Group, VIB-Department of Molecular Genetics, University of Antwerpen, Antwerpen, Belgium; 12 Department of Neurology, University Hospital of Antwerpen, Antwerpen, Belgium; 13 Institute of Neuropathology, Heinrich-Heine-University Düsseldorf, Düsseldorf, Germany; 14 Department of Cardiology, Angiology and Pneumology, University Hospital Heidelberg, Heidelberg, Germany; 15 DZHK (German Centre for Cardiovascular Research), partner site Heidelberg, Heidelberg, Germany; 16 Department of Neurology, Martin-Luther-University Halle-Wittenberg, Halle, Germany; 17 Institute of Neuropathology, Klinikum Bremen-Mitte, Bremen, Germany; 18 JARA - Translational Brain Medicine, Jülich and Aachen, Germany

**Keywords:** MFM, Next generation sequencing, bcl-2 associated athanogene protein 3, Protein aggregation, Hearing impairment, Polyneuropathy

## Abstract

**Background:**

Myofibrillar myopathies (MFM) are a group of phenotypically and genetically heterogeneous neuromuscular disorders, which are characterized by protein aggregations in muscle fibres and can be associated with multisystemic involvement.

**Methods:**

We screened a large cohort of 38 index patients with MFM for mutations in the nine thus far known causative genes using Sanger and next generation sequencing (NGS). We studied the clinical and histopathological characteristics in 38 index patients and five additional relatives (n = 43) and particularly focused on the associated multisystemic symptoms.

**Results:**

We identified 14 heterozygous mutations (diagnostic yield of 37%), among them the novel p.Pro209Gln mutation in the *BAG3* gene, which was associated with onset in adulthood, a mild phenotype and an axonal sensorimotor polyneuropathy, in the absence of giant axons at the nerve biopsy. We revealed several novel clinical phenotypes and unusual multisystemic presentations with previously described mutations: hearing impairment with a *FLNC* mutation, dysphonia with a mutation in *DES* and the first patient with a *FLNC* mutation presenting respiratory insufficiency as the initial symptom. Moreover, we described for the first time respiratory insufficiency occurring in a patient with the p.Gly154Ser mutation in *CRYAB*. Interestingly, we detected a polyneuropathy in 28% of the MFM patients, including a *BAG3* and a *MYOT* case, and hearing impairment in 13%, including one patient with a *FLNC* mutation and two with mutations in the *DES* gene. In four index patients with a mutation in one of the MFM genes, typical histological findings were only identified at the ultrastructural level (29%).

**Conclusions:**

We conclude that extraskeletal symptoms frequently occur in MFM, particularly cardiac and respiratory involvement, polyneuropathy and/or deafness. *BAG3* mutations should be considered even in cases with a mild phenotype or an adult onset. We identified a genetic defect in one of the known genes in less than half of the MFM patients, indicating that more causative genes are still to be found. Next generation sequencing techniques should be helpful in achieving this aim.

## Background

Myofibrillar myopathies (MFM) are a group of phenotypically and genetically heterogeneous neuromuscular disorders. The morphological hallmark of MFM is the presence of protein aggregations in muscle fibres, a focal myofibrillar disorganisation starting at the Z-disk and an ectopic expression of proteins, such as desmin [1]-[6].

Muscle biopsies of MFM patients reveal protein aggregates that are dark blue or purple on the modified Gomori trichrome (mGT) and pink on the haematoxylin and eosin (HE) stains. The aggregations are lacking oxidative enzyme activity on the nicotinamide adenine dinucleotide tetrazolium reductase (NADH-TR) stains. Other light microscopic findings in MFM are rimmed and non-rimmed vacuoles and sometimes cytoplasmic bodies. At the ultrastructural level, the combination of Z-disk streaming and distinct types of protein accumulations is characteristic for MFM and can even give hints towards the mutated gene [7].

To date, mutations in nine genes are known to cause MFM: desmin (*DES*) [8], αB-crystallin (*CRYAB*) [9], myotilin (*MYOT,* also *TTID*) [10], filamin C (*FLNC*) [11], Z-band alternatively spliced PDZ motif-containing protein (*ZASP,* also *LDB3*) [12], four and a half LIM domain protein 1 (*FHL1*) [13], bcl-2 associated athanogene protein 3 (*BAG3*) [14], dnaJ homolog subfamily B member 6 (*DNAJB6*) [15] and titin (*TTN*) [16]. Mutations in exon 343 of the A-band region of *TTN* were discovered to cause MFM only very recently [16]. Inheritance in MFM is autosomal dominant in most patients, autosomal recessive in rare cases and X-linked in patients with mutations in *FHL1*. A broad inter- and intrafamilial variability of phenotypes can be seen in MFM. The age at onset of MFM depends on the underlying gene, mutation and inheritance pattern, but usually the disease starts in adulthood [17],[18]. Nevertheless also childhood onsets have been described [8],[13],[14],[19]-[21]. The initial symptom in MFM most frequently is skeletal muscle weakness, other muscular symptoms such as atrophy, hypertrophy, muscle rigidity, contractures, pain or cramps can be present [13],[22]-[26]. Also, the respiratory muscles can be affected in MFM, resulting in a restrictive respiratory insufficiency, which has been reported for most MFM subtypes [4],[8],[11],[14],[27]-[31].

Furthermore, MFM can be associated with distinct multisystemic symptoms, the most frequent being cardiac involvement, ranging from conduction defects and arrhythmias to cardiomyopathies and sudden cardiac death [4],[9],[15],[26],[29],[30],[32]-[38]. The smooth muscles can also be affected in MFM, presenting as chronic diarrhoea or enteric hypomotility [37],[39]-[42], or contributing to bulbar symptoms like swallowing difficulties [27],[28],[31],[33],[37],[43]-[45]. Further symptoms associated with MFM exceed the muscles, such as polyneuropathy (PNP) [10]-[12],[14],[46], cataracts [9],[31],[33], gynaecomastia [29],[47], and hearing impairment [39],[40],[48],[49].

Serum creatine kinase levels (CK) in MFM patients are normal or only slightly elevated. Electromyography (EMG) reveals myopathic or mixed patterns, but also (pseudo-) myotonic discharges can appear [17],[50]. Magnetic resonance imaging (MRI) of the muscles plays an increasing role in the differential diagnosis of muscular disorders and of MFM in particular [51],[52].

In the present study, we screened a large cohort of 38 unrelated index patients with MFM for mutations in the nine causative genes, employing next generation sequencing (NGS) and traditional Sanger sequencing. We identified a mutation in 14 index patients, including one novel *BAG3* mutation. We performed a detailed clinicopathological characterisation in the index patients and five affected relatives and particularly focused on the associated multisystemic symptoms. We highlight new phenotypical findings in MFM and correlate our data with the literature.

## Patients and methods

### Patients

**Figure 1:**
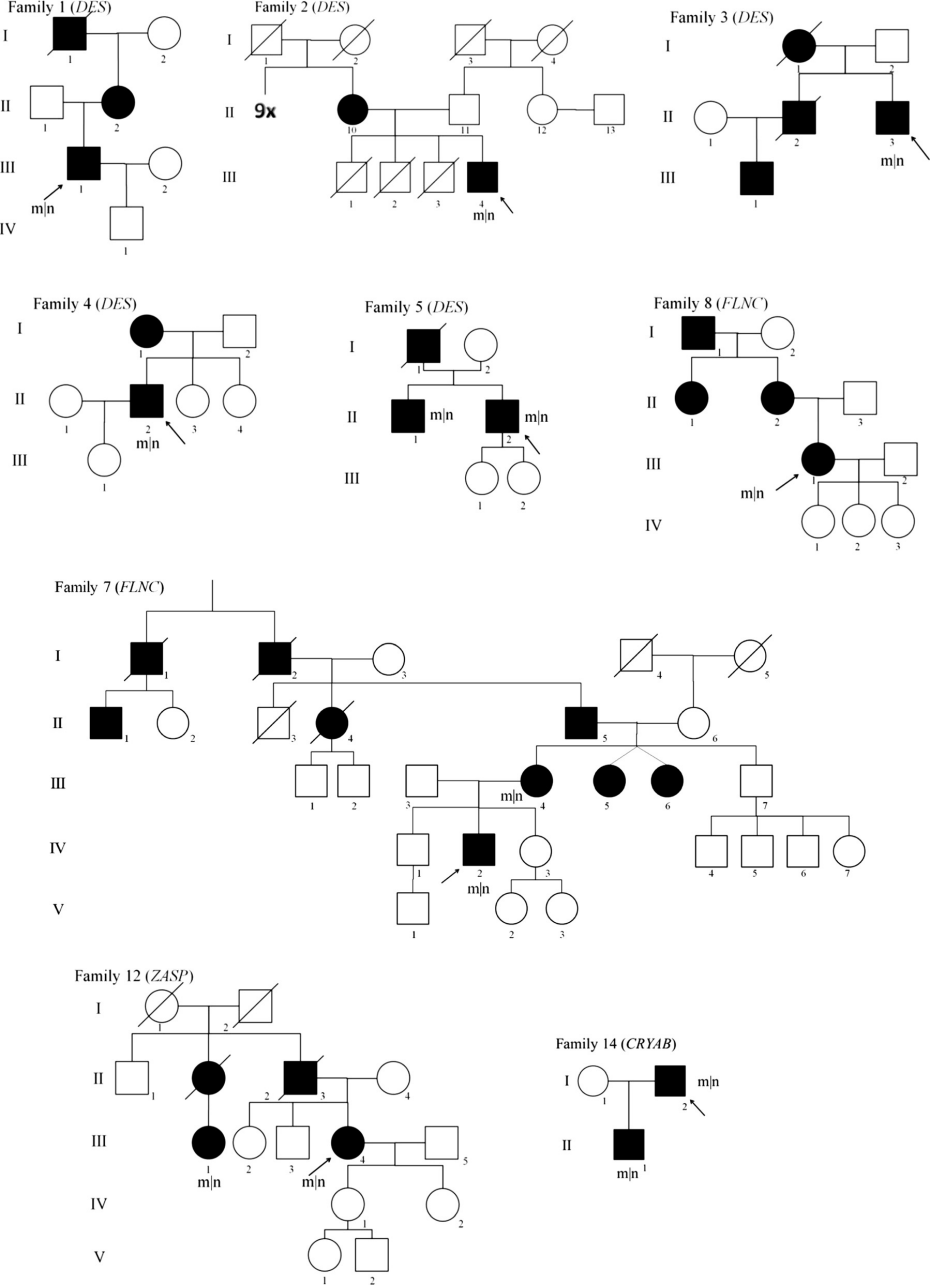
**Pedigrees of MFM patients with a confirmed mutation and autosomal dominant inheritance.** Index patients are indicated by an arrow; m|n, mutated allele| wildtype allele, indicating all patients who are genetically confirmed heterozygous mutation carriers.

In this study, we included 43 patients with MFM belonging to 38 unrelated families (Figure 1). We selected the MFM cases from the muscle and nerve biopsy archive at the Institute of Neuropathology, and from the Neuromuscular Clinic at the Department of Neurology, at the RWTH Aachen University Hospital (Aachen, Germany). The biopsy of patient F10.1 was also included in the study of Joshi et al. [53]. Additionally, eight index patients/biopsies with MFM were provided by referring centres. The study was performed according to the Declaration of Helsinki and was approved by the ethical committee of the RWTH Aachen University. Written informed consent was obtained from all patients.

### Clinical and paraclinical evaluation

In all 43 patients, we performed a detailed neurological examination, including hearing and swallowing tests, and a blood analysis of the creatine kinase (CK) level. Nerve conduction velocity (NCV) and electromyography (EMG) studies, heart (electrocardiogram (ECG), echocardiography, long-term ECG) and lung examinations (spirometry/bodyplethysmography) were performed in the majority of patients. In addition, an ophthalmological exam was undertaken in patients with complaints of ocular involvement. An EMG was done in all patients with an identified gene defect, except for patients F5.II.2 and F14.1, a diagnostic muscle MRI of the lower extremities in four patients (F1.III.1, F2.III.4, F10.1, F12.III.3) and a whole-body muscle MRI in one (F7.III.4) (1.5 Tesla, Philips, Intera, Best – the Netherlands).

### Light microscopy (LM) and electron microscopy (EM) of muscle and nerve biopsies

Open muscle biopsies had been obtained from the 38 index patients for diagnostic purposes after written informed consent. The muscle tissue sample was divided into unfixed (cryostat), formaldehyde-fixed (paraffin) and glutaraldehyde-fixed (EM) sections and further prepared according to routine protocols. Standard enzyme histochemical and immunohistochemical stains were applied [54]. For electron microscopy (EM) of muscle tissue, which was performed in 31 out of 38 probands, we used a CM10 transmission electron microscope (Philips, Amsterdam, the Netherlands). In addition, seven index patients had simultaneously a diagnostic sural nerve biopsy taken, among them patient F9.1 and F13.1 with a confirmed mutation and a polyneuropathy. The nerve biopsies were processed following standard procedures [55].

### Genetic analyses

Peripheral blood samples and a written informed consent for genetic analysis were available from the 38 index patients, as well as from the affected mother of patient F7.IV.2, the affected brother of patient F5.II.2, the cousin of patient F12.III.4 and the son of patient F14.1. Genomic DNA was extracted using standard procedures. We used two different techniques for genetic analyses in our cohort: Sanger sequencing [56] and next generation sequencing (NGS) [57].

### Sanger sequencing of the MFM genes

We had started screening a group of 21 MFM index patients with conventional Sanger sequencing before the NGS panel for MFM was established. We analysed *DES*, *CRYAB*, *MYOT* (exon 2 and 9), *FLNC* (exon 48), *ZASP* (exon 4–10), *FHL1*, *BAG3* and *DNAJB6*. We performed PCR using standard protocols and used the BigDye Terminator v1.1 mix (Applied Biosystems) for cycle sequencing reactions according to the instructions in the kit. Sequence data were generated with an ABI 3130xl capillary sequencer (Applied Biosystems, Carlsbad, CA, USA) using standard protocols and analysed by GenSearch v.4.0.8 (PhenoSystems, Lillois, Belgium).

### NGS panel diagnostics in MFM

In a NGS approach, the coding exons of all causative genes with the exception of the most recently identified *TTN* gene were sequenced in the additional 17 index patients included in our study: *DES* (exon 1–9, NM_001927.3), *MYOT* (exon 2–10, NM_006790.2), *ZASP* (exons 5–9 of NM_001080116.1 and exons 4, 7, 8 and 10 of NM_007078.2), *FHL1* (exon 3–8, NM_001159702.2), *BAG3* (exon 1–4, NM_004281.3), *DNAJB6* (exon 2–8, NM_005494.2), *CRYAB* (exon 1–3, NM_001885.1) and *FLNC* (exon 1–48, NM_001458.3). Furthermore, in 10 index patients of our first cohort in which Sanger sequencing was initially performed (see above) and in which no mutations were identified, we subsequently analysed the complete *FLNC* gene (exon 1–48) applying NGS. Target enrichment was performed with the Access Array System (Fluidigm Corporation, South San Francisco, USA) followed by emulsion PCR and sequence analyses on a GS Junior using kits and instructions by Fluidigm and 454 Life Sciences (Branford, USA). Sequence data were analysed with GenSearch NGS v.1.4 (PhenoSystems, Lillois, Belgium) automatically aligning data to the reference sequences mentioned above. Variants within exons and 20 bp of the flanking intronic sequences were detected with VarScan (http://varscan.sourceforge.net/) using following filters: coverage >15, frequency >20% for heterozygous or >75% for homo- and hemizygous variants. Candidate variants were verified by Sanger sequencing on an ABI 3130xl automated capillary sequencer using standard protocols, and their effect predicted with bioinformatic tools like PolyPhen-2 (http://genetics.bwh.harvard.edu/pph2/index.shtml), SIFT (http://sift.jcvi.org/) and MutationTaster (http://www.mutationtaster.org/) provided by Alamut (Interactive Biosoftware, Rouen, France).

### Sanger sequencing of exon 343 of *TTN*

Finally, we performed Sanger sequencing to analyse exon 343 of *TTN* in 25 of our index patients, in whom an underlying gene defect had thus far not been identified (21 patients) or only an unknown variant was revealed (4 patients). PCR, sequencing reaction and data analysis of exon 343 of the *TTN* gene (NM 001267550.1) have been performed using standard protocols as described above.

## Results

### Genetic results in our MFM cohort (n = 43): known and novel pathogenic mutations

In 14 index patients, we identified a heterozygous pathogenic mutation in one of the nine genes causing MFM (Table 1), corresponding to a diagnostic yield of 37% in our MFM cohort. In ten patients we found the underlying gene defect using the initial Sanger sequencing study and in four patients by directly employing NGS panel diagnostics. Nine out of the 14 index patients had a positive family history corresponding with an autosomal dominant inheritance (for pedigrees see Figure 1), whereas the others occurred sporadically (Patient F6.1 with a *DES* mutation, Patient F13.1 with a mutation in *BAG3* and all three patients, F9.1, F10.1 and F11.1, with a *MYOT* mutation, Table 1). We identified six heterozygous mutations in *DES*, four of these were the Arg350Pro mutation, two different mutations in *FLNC*, and three *MYOT* mutations, including two times the Ser60Phe mutation in exon 2. One mutation each was found in *ZASP*, *CRYAB* and in *BAG3* (Table 1, Figure 1). The mutation in *BAG3* represents a novel mutation, whereas the other mutations have been described previously.

**Table 1:** Clinical characteristics of myofibrillar myopathy patients with an identified mutation

**Pat.**	**M/F**	**FH**	**Gene**	**Exon**	**Mutation**	**AAO**	**AAE**	**First symptom**	**Weakness**										**Multisystemic symptoms**			**CK**
									**UL prox.**	**UL dist.**	**LL prox.**	**LL dist.**	**ptosis**	**axial**	**scapular winging**	**dys-phagia**	**dys-phonia**	**respiratory involvemen**t	**cardiac involvement**	**PNP**	**hearing imp.**	
**F1.III.1**	M	+	*DES*	6	Arg350Pro	39	47	weakness LL	+	-	+	+	-	-	-	-	-	-	-	-	-	1031
**F2.III.4**	M	+	*DES*	6	Arg350Pro	42	74	weakness LL	-	-	+	+	-	+	-	-	-	-	-	-	-	2690
**F3.II.3**	M	+	*DES*	6	Arg350Pro	48	59	dyspnoea	+	+	+	+	+	-	-	-	-	VC 45%,NV	TAA intermittent, LV hypertrophy	-	+	2044
**F4.II.2**	M	+	*DES*	6	Arg350Pro	36	42	weakness LL	+	-	+	+	-	-	-	+	-	-	-	-	+	1159
**F5.II.2**	M	+	*DES*	1	Ser2Ile	60	72	syncopes	+	-	+	+	-	+	+	-	-	-	3°AVB, PM	-	-	nl
**F6.1**	F	-	*DES*	3	Glu245Asp	51	62	weakness LL	-	-	+	+	-	-	-	-	-	-	DCM, AF, 3 °AVB, PM, TI	-	-	nl
**F7.IV.2**	M	+	*FLNC*	18	Val930_Thr 933del	28	40	weakness LL	-	-	+	-	-	-	+	-	-	-	-	-	-	629
**F7.III.4**	F	+	*FLNC*	18	Val930_Thr 933del	53	60	weakness LL	-	-	+	+	-	-	-	-	-	-	-	-	+	388
**F8.III.3**	F	+	*FLNC*	48	Trp2710X	53	59	dyspnoea	+	-	+	+	-	-	+	-	-	VC 35%,NV	-	-	-	500
**F9.1**	M	-	*MYOT*	2	Ser60Phe	67	71	dyspnoea	-	-	-	+	+	-	-	-	-	-	LBBB, HF, AF, 3°AVB intermittent, PM	ad/sm	-	206
**F10.1**	M	-	*MYOT*	2	Ser60Phe	63	67	myalgia LL	-	-	+	-	-	-	-	-	-	-	-	-	-	589
**F11.1**	M	-	*MYOT*	2	Ser55Phe	55	60	weakness LL	+	-	+	+	-	-	-	-	-	mild	AF, PM, ICD, DCM, HTx,	-	-	608
**F12.III.3**	F	+	*ZASP*	6	Ala165Val	46	57	walking difficulties	-	+	-	-	-	-	-	-	-	-	-	-	-	182
**F13.1**	M	-	*BAG3*	3	Pro209Gln	34	43	weakness LL	+	-	+	+	-	-	+	-	-	-	-	a/sm	-	1050
**F14.1**	M	+	*CRYAB*	3	Gly154Ser	69	69	rhabdomyolysis	-	-	-	-	-	-	-	-	-	VC 65%	-	-	-	2000

M/F, male/female; FH, family history; AAO, age at onset; AAE, age at examination; CK, level of creatine kinase in serum in U/L (normal value < 174); UL, upper limb; LL, lower limb; prox., proximal; dist., distal; PNP, polyneuropathy; Hearing imp., hearing impairment; VC, vital capacity; NC, nocturnal ventilation; TAA, tachyarrhythmia absoluta; LV, left ventricle; 3° AVB, complete atrioventricular conduction block; nl, normal; DCM, dilated cardiomyopathy; AF, atrial fibrillation; PM, pace maker; TI, tricuspid insufficiency; LBBB, left bundle branch block; HF, heart failure; ad/sm, axonal demyelinating/sensorimotor; ICD, implantable cardioverter defibrillator; HTx, heart transplantation; a/sm, axonal/sensorimotor; for abbreviations of genes see text.

The c.626C > A, p.Pro209Gln mutation in exon 3 of *BAG3* (Patient F13.1, Table 1) is so far not known in the databases of the NHLBI exome sequencing project (ESP) or the 1000 genome project and was predicted to be disease causing by all used prediction programmes. Further arguments for pathogenicity came from familial segregation analysis, in which both healthy parents did not harbour the mutation, indicating a *de novo* mutation.

### Genetic results in our MFM cohort (n = 43): polymorphisms and unclassified variants

With means of NGS panel diagnostics we found a large number of variants, especially in *FLNC*. The suspicious variants with a minor allele frequency beneath 1% are listed in Additional file 1. We assessed these variants with various databases and prediction programmes. Especially the pathogenicity of the c.6595G > A, p.Gly2199Arg mutation in exon 40 of *FLNC* (patient F20.1, Additional file 1) finally remained unclear. This genetic variant has only been counted once by the NHLBI exome sequencing project (ESP) and was predicted to be benign by PolyPhen-2, but disease causing by Mutation Taster. The glycin residue at position c.6595 is highly conserved, as well as the nucleotide at this position. Unfixed muscle tissue of this patient was not available anymore and no new muscle biopsy could be obtained, so that additional proteomic analyses for possible further clarification could not be performed. Moreover, family members were not available for segregation analysis to further clarify the pathogenicity.

### Clinical findings in MFM patients with an identified gene defect (n = 14)

The 14 index patients with an identified mutation included eleven males and three females (Table 1). The age at onset ranged from 28 to 69 years (mean 49 years). The most common initial symptom was weakness in the legs (64%). In three patients the first symptom was dyspnoea (21%) and in one syncopes (7%), indicating a respiratory or cardiac onset of the disease. At the time of examination, the disease persisted already up to 32 years (mean disease duration 9 years). Muscle weakness was distributed in the distal and proximal lower limbs, combined with weakness in the proximal upper limbs in six of the 14 patients. Patients F3.II.3 and F6.1 were wheelchair bound since the age of 59 and 60 years respectively, both after 11 years of disease progression. Scapular winging was evident in 29% of the patients, 14% showed additional axial weakness and 14% a ptosis without ophthalmoparesis or facial weakness (Table 1). In 64% of the patients, atrophy of the distal lower limbs was present, among them the two patients with an additional PNP. EMG revealed a myopathic pattern in 58% and a mixed pattern in 42%, in three of the patients with a mixed pattern also pseudomyotonic discharges were present. The serum CK levels varied from normal to 16 fold elevated (mean: 5.5 N) (Table 1). Only patient F14.1 did not show permanent skeletal muscle weakness.

### Multisystemic symptoms in the whole MFM cohort (n = 43)

In general, 16% of the patients included in the study (n = 43) had respiratory involvement and 60% presented one or more multisystemic symptom(s) (Table 2). The most striking finding was the large number of MFM patients with mainly a sensorimotor axonal-demyelinating polyneuropathy (12/43 or 28%), which occurred as frequently as cardiac disease (Table 2). In 50% (n = 6) of the MFM patients with a polyneuropathy, no other possible cause was present. However, another 33% (n = 4) suffered from diabetes mellitus and 17% (n = 2) had taken neurotoxic medications, as possible (additional) causes of a polyneuropathy. The PNP was diagnosed after 0–6 years of disease duration (mean: 1.7 years). 14% of the MFM patients suffered from hearing impairment, with a symptom onset between 16 and 80 years of age. Half of these patients used hearing devices. We did not detect diarrhoea, intestinal malabsorption or pseudoobstruction in our cohort.

**Table 2:** Multisystemic symptoms of all included MFM patients (n = 43)

**Symptom**		**Frequency**	
**Respiratory symptoms (6)**	restricted vital capacity	6**	(1x *DES*, 1x *FLNC,* 1x *MYOT,* 1x *CRYAB*)
	ventilation assistance	1	
	nocturnal ventilation	2	(1x *DES*, 1x *FLNC*)
**Cardiac symptoms (12)**	atrial fibrillation	8	(1x *DES*, 2x *MYOT*)
	tachyarrhythmia absoluta	1	(1x *DES*)
	ventricular tachycardia	1	
	bradycardia	1	
	ventricular extrasystoles	1	
	3° AV block	3*	(2x *DES*, 1x *MYOT*)
	LBBB	2	(1x *MYOT*)
	RBBB	1	
	bifascicular block	1	
	tricuspid insufficiency	2	(1x *DES*)
	aortic/mitral/tricuspid insufficiency	1	
	heart failure	3	(1x *MYOT*)
	LV hypertrophy	3	
	DCM	2	(1x *DES*, 1x *MYOT*)
	pace maker	6	(2x *DES*, 2x*MYOT*)
	ICD	1	(1x *MYOT*)
	heart transplantation	1	(1x *MYOT*)
	sudden cardiac death	0	
**Polyneuropathy (12)**	axonal	3	(1x *BAG3*)
	demyelinating	0	
	axonal-demyelinating	9	(1x *MYOT*)
	sensory	0	
	sensory + autonomic	1	
	motor	1	
	sensorimotor	10	(1x *MYOT*, 1x *BAG3*)
**Bulbar symptoms (8)**	dysphagia	5	(1x *DES*)
	dysphonia	2	(1x *DES*)
**Hearing impairment**		6	(2x *DES*, 1x *FLNC*)
**Gynaecomastia**		1	

One patient can present more than one multisystemic symptom; between brackets, the number of cases with an identified mutation in whom the symptom was present; **, symptom was the onset symptom in two cases; 3° AV block, complete atrioventricular conduction block; *, symptom was the onset symptom in one case; LBBB, left bundle branch block; RBBB, right bundle branch block; LV, left ventricle; DCM, dilated cardiomyopathy; ICD, implantable cardioverter defibrillator.

### Multisystemic symptoms in MFM patients with an identified mutation (n = 14 and affected relatives)

In the group of index patients with a pathogenic mutation (n = 14) 64% showed at least one multisystemic symptom (Figure 1, Tables 1 and 2). Patient F3.II.3 harbours the Arg350Pro mutation in *DES* and presented with exertional dyspnoea as initial symptom at 48 years of age. Subsequently, he developed dyspnoea at rest, had a vital capacity (VC) of 45% of the theoretical value and needed non-invasive continuous positive airway pressure (CPAP) ventilation at night. Furthermore, he presented an intermittent tachyarrhythmia absoluta and a hypertrophic left ventricle at echocardiography. He also suffered from bilateral hypacusis and used a hearing aid on the right side since the age of 52 years.

Patient F4.II.2 carried the same Arg350Pro mutation in *DES* and recognised swallowing problems after six years of disease progression. He also presented a bilateral hypacusis since the age of 38 years.

Only the affected mothers of two more patients with the Arg350Pro mutation in *DES* (patient F1.III.1; F2.III.4) showed multisystemic symptoms (respiratory insufficiency, cardiac involvement with a pace maker implantation, polyneuropathy).

Patient F6.1 carried the Glu245Asp mutation in *DES* and exhibited cardiac symptoms: a dilated cardiomyopathy was diagnosed approximately ten years after disease onset and a chronic atrial fibrillation and complete atrioventricular block occurred, necessitating pace maker implantation.

The patient carrying the Ser2Ile mutation in *DES* (patient F5.II.2) presented with syncopes as the first symptom at the age of 60 years, due to a complete atrioventricular conduction block leading to pace maker implantation. At the age of 65 years, he developed mild distal lower leg weakness and dysphonia. Other causes of dysphonia were excluded. Interestingly, the patient’s father also had dysphonia and a pacemaker implantation at the age of 58 years. The patient’s brother, carrying the same mutation, received a pacemaker at the age of 45 years and had mild distal involvement of the lower legs, but no dysphonia.

Patient F8.III.3 with the Trp2710X mutation in *FLNC* developed respiratory problems three years before weakness in the extremities occurred. After five years of disease progression, the patient was dependent on non-invasive nocturnal ventilation. VC decreased to 35%.

The affected mother of the index patient harbouring the Val930_Thr933del mutation in *FLNC* (patient F7.III.4) presented with bilateral hearing impairment and hearing aids at the age of 60 years.

Patient F9.1 carrying the Ser60Phe mutation in *MYOT* developed exertional dyspnoea due to heart failure at the age of 64 years as the first symptom. A left bundle branch block, an intermittent atrioventricular conduction block and atrial fibrillation were diagnosed, and a pace maker was implanted. The patient also suffered from coronary disease. Four years later, a sensorimotor axonal-demyelinating polyneuropathy was diagnosed.

In Patient F11.1 with a Ser55Phe mutation in *MYOT*, cardiac symptoms began shortly after skeletal weakness became apparent at the age of 55 years. He had a permanent atrial fibrillation with tachyarrhythmia absoluta and received an implantable cardioverter defibrillator (ICD). In addition, he had several myocardial infarctions due to coronary disease. One year later, a dilated cardiomyopathy was diagnosed and heart transplantation was performed.

Patient F14.1 carries the p.Gly154Ser mutation in *CRYAB* and presented at the age of 69 years with several episodes of rhabdomyolysis. The patient also complained about exercise intolerance and fatigue. The neurological examination was normal, but a respiratory involvement with a VC of 65% in lying position was diagnosed. His 42-year-old son, carrying the same mutation, was asymptomatic and only showed a moderate serum CK elevation.

### MRI findings in MFM patients with an identified mutation

Muscle MRI findings are summarised and compared to the literature in Table 3.

**Table 3:** MRI findings in five MFM patients with an identified mutation

	**Fischer et al. 2008**				**Current study**				
**Gene**	***DES***	***FLNC***	***MYOT***	ZASP*	***DES***		***FLNC***	***MYOT***	***ZASP***
**Patient**					**F1.III.1**	**F2.III.4**	**F7.III.4**	**F10.1**	**F12.III.3**
**Pelvic level**									
Gluteus maximus	2,3	1,3	2	3	3	np	1	np	np
Gluteus medius	1,8	1,7	2,8	1	1	np	1	np	np
Gluteus minimus	1,8	1,7	3,3	3	3	np	1	np	np
**Mid thigh level**									
Vastus lateralis	1,2	1,2	1,6	2	3	1	2	1	1
Vastus intermedius	1,2	2,8	2,8	3	4	1	3	1	1
Vastus medialis	1,3	2,3	2,8	3	4	1	3	1	1
Rectus femoris	1,3	0,4	0,1	2	1	1	2	1	1
Sartorius	2,8	0,7	1,5	1	4	3	1	2	1
Gracilis	2,2	0,6	0,4	1	3	3	1	1	1
Biceps femoris	1,5	3,3	2,9	4	1	1	3	3	3
Adductor magnus	1,7	3,2	3,2	4	3	1	3	3	2
Semitendinosus	3,1	1,8	1,2	3	3	3	3	2	1
Semimembranosus	1,5	3,3	2,8	4	2	1	3	2	3
**Mid leg level**									
Tibialis anterior	1,9	2,8	3,1	3	3	2	0	1	3
Peroneal muscle group	3	2,7	2,8	3	2	4	1	1	2
Medial gastrocnemius	2	3	3,4	3	2	3	3	4	4
Lateral gastrocnemius	2,1	0,9	2,1	3	2	3	1	3	4
Soleus	2,2	3,6	3,9	4	4	2	3	3	4

5-point scale according to Fischer et al. 2008 [51]: stage 0 is referred to a normal muscle appearance; stage 1 means traces of increased signal intensity on the T1-weighted MR sequences; stage 2 refers to increased signal intensity on MRI in less than 50% of the muscle; stage 3 means an increased signal intensity on MRI in more than 50% of the muscle; stage 4 indicates an increased signal intensity on MRI in the entire muscle (end-stage disease); *, *ZASP* classification is based on Figure four in the study of Fischer et al. 2008; np, not performed; for abbreviations of genes see text.

### Histological findings of the MFM cohort

In the 14 index patients with an identified mutation we consistently found an increased variability of fibre diameter (100%), frequent nuclear bags (64%), internal nuclei (86%), vacuoles (64%), rimmed vacuoles (50%) and increased endomysial connective tissue (79%). In 43% of the biopsies, cytoplasmic bodies and core-like lesions on the NADH-TR staining were revealed. Myofibrillar disorganisation was seen in all cases, but characteristic protein aggregations in the cytoplasm of mGT stained fibres were found in only 10 of the 14 cases (71%) (Patient F11.1: in the vastus medialis muscle biopsy protein aggregations were seen, but not in the simultaneously obtained tibialis anterior muscle biopsy). So 29% only showed typical MFM findings at the ultrastructural level (EM was performed in 11 of the 14 cases). Z-disk streaming and myofibrillar disorganisation were seen in 100%, granulofilamentous material in 64%. Abnormal mitochondria (64%), tubulofilamentous accumulations (29%) and cytoplasmic bodies (36%) were other frequent findings. Immunhistochemistry with antibodies directed against desmin showed immunoreactivity in all 8 performed cases.

In the group of 24 patients, in whom the underlying genetic defect had not been identified, we made similar observations. Characteristic protein aggregations in the cytoplasm of mGT stained fibres were found in 76% of the cases, myofibrillar disorganisation in 82%.

In ten of the 12 MFM patients additionally presenting a polyneuropathy, we observed neurogenic muscle atrophy, which found expression in angular shaped muscle fibres and fibre type grouping.

### Clinicopathological phenotype of the patient with a novel *BAG3* mutation

Patient F13.1 harbours the novel Pro209Gln mutation in *BAG3* (Table 1)*.* He presented first symptoms of distal lower limb weakness and symmetrical calve atrophy at the age of 34 years. The skeletal muscle weakness spread to the proximal lower limbs and finally to the proximal upper limbs with scapular winging after 2 more years of disease progression. Moreover, he developed an axonal sensorimotor polyneuropathy, as multisystemic symptom of MFM. No other obvious cause for the PNP could be found. The PNP was first diagnosed at the age of 39 years and finds expression in decreased vibration sense and ataxic, clumsy gait as well as an increased sensitivity for cold. EMG showed a mixed pattern and the maximum CK level was 1050 U/L (Table 1). The muscle biopsy showed desmin positive protein deposits, vacuoles and core-like lesions as well as some necrotic fibres. At the ultrastructural level, tubulofilamentous accumulations, lobulated nuclei and glycogen accumulations were seen, in addition to typical MFM findings like Z-disk streaming and the accumulation of granulofilamentous material. We did not observe regenerating fibres or apoptotic nuclei. The ultrastructural study of nerve tissue revealed no giant axons.

## Discussion

**Figure 2:**
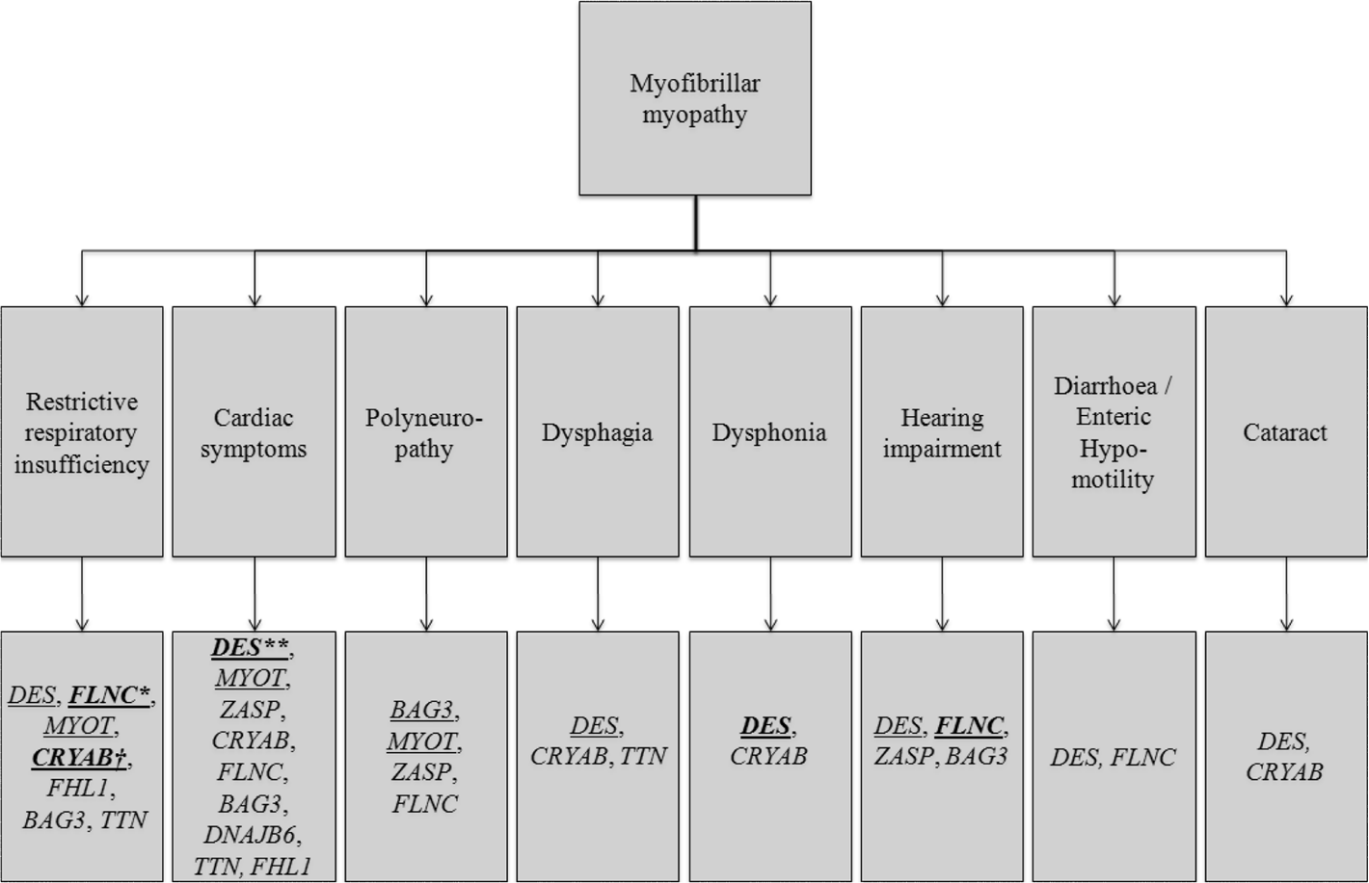
**Flowchart of multisystemic symptoms of MFM.** Underlined, findings in current study; bold, new findings in current study; *, for the first time described as onset symptom with *FLNC* mutation in current study; **, for the first time described as onset symptom with p.Ser2Ile *DES* mutation in current study; † for the first time described with p.Gly154Ser *CRYAB* mutation in our study; not underlined, literature findings.

In a large cohort of MFM patients, we identified heterozygous mutations in 14 of 38 index patients (diagnostic yield of 37%), including the novel p.Pro209Gln mutation in exon 3 of *BAG3*, using Sanger and next generation sequencing (NGS) of the nine thus far known causative genes of MFM. Furthermore, we discovered new phenotypes associated with previously described mutations, such as hearing impairment with a *FLNC* mutation, dysphonia with a mutation in *DES* and the first patient with a *FLNC* mutation presenting respiratory insufficiency as the onset symptom. Interestingly, we detected a polyneuropathy in more than a quarter of the MFM patients, including a *BAG3* and a *MYOT* case (Figure 2).

### Methodological issue: Sanger versus NGS sequencing

In ten of our patients, a mutation in one of the MFM-causative genes was identified using conventional Sanger sequencing. By employing NGS, we additionally found four mutations, among them the novel *BAG3* mutation, as well as a lot of variants, especially in *FLNC*, beyond the mutational hotspot in exon 48. The mass of variants that are found using NGS and their sometimes difficult interpretation can currently still be considered as a drawback of this technique. However, knowledge concerning the variants will increase in the following years as NGS will become more widely available and databases more complete. Advantages of NGS are reduced costs and decreased time to diagnosis. Furthermore, a specific and often unreliable genetic testing sequence is made redundant, especially in diseases with overlapping morphological and/or clinical phenotypes, such as MFM. The difficulty in interpretation of certain variants is illustrated by the unknown variant p.Gly2199Arg in exon 40 of *FLNC* of patient F20.1 (Additional file 1).

### Novel mutation identified in *BAG3* and associated phenotype

The novel *BAG3* mutation is located at the amino acid position p.209 and proline is replaced by glutamine. At the same position the p.Pro209Leu mutation is situated, which is the most common *BAG3* mutation associated with MFM [14],[36],[46],[48]. In all previously described MFM cases with the p.Pro209Leu mutation, the disease started within the first or second decade and was hallmarked by rapidly progressive muscle weakness, severe cardiac and respiratory involvement and early death of the patients in some cases. In nine of the eleven reported cases an axonal polyneuropathy was additionally found. Our patient’s (Patient F13.1, Table 1) mild phenotype with adult-onset skeletal muscle weakness and the absence of cardiac or respiratory involvement clearly differs from the other *BAG3*-associated MFM cases, solely his axonal sensorimotor PNP is in concordance. Only one other adult-onset patient with *BAG3*-related MFM has thus far been described: a woman harbouring the p.261-265 RAASP deletion in *BAG3*, who showed involvement of the orbicularis oculi muscles and the lower limb muscles, as well as photophobia and diplopia, starting at the age of 60 years. Her muscle biopsy revealed typical MFM findings [58].

In our patient’s nerve and muscle biopsy no giant axons could be found, which were described before in *BAG3*-associated MFM cases [36],[46],[48]. Muscle biopsy findings were typical for MFM. We observed some lobulated nuclei at electron microscopy, corresponding to the findings of Selcen et al. who also described abnormal nuclei up to apoptotic changes in one MFM patient with a *BAG3* mutation [14].

### Multisystemic symptoms in MFM, unusual phenotypes

We identified new phenotypes considering the multisystemic symptoms of our patients in association with certain mutations in the MFM genes (Figure 2; for review of the literature see Additional file 2). We reported hearing impairment for the first time in patients with the p.Arg350Pro *DES* mutation and for the first time in combination with a *FLNC* mutation (p.Val930_Thr933del). Also three other patients of our cohort (Table 2) presented with hearing problems, in one case (unidentified gene defect) the complaints began at the age of 16 years. Hearing impairment as a multisystemic symptom of MFM was described by Kraya et al. in two patients with a *ZASP* mutation, in a patient harbouring an unknown variant in *BAG3* and in one autosomal recessive *DES* case, as a congenital feature [39],[40],[48],[49].

We moreover present two new onset symptoms: syncopes due to a conduction block associated with the p.Ser2Ile mutation in *DES* and dyspnoea due to respiratory insufficiency for the first time with a *FLNC* mutation. In the patient with the *DES* mutation mild skeletal muscle involvement appeared only after several years. The two reported cases with the same mutation both showed skeletal muscle involvement at baseline [59], so this is the first described pure cardiac onset case associated with this mutation. Moreover dysphonia was noted in the propositus and his father. So far, only dysphagia and nasal voice have been described with *DES* mutations [27], dysphonia has only been described with *CRYAB* mutations [31].

Respiratory insufficiency has been described in association with the p.Trp2710X mutation in *FLNC*, and frequently was the cause of death, but never the initial symptom [11],[30],[60]. It was not reported as an initial symptom in patients with other *FLNC* mutations, either. Also in patient F14.1 respiratory involvement was diagnosed, which has not been reported before with the p.Gly154Ser mutation in *CRYAB*[47],[61].

The high frequency of polyneuropathy in our MFM cohort was striking (Table 2). We found PNP in combination with a novel *BAG3* and a known *MYOT* (p.Ser60Phe) mutation (Table 1). Both, *BAG3* and *MYOT* mutations, have been reported in MFM patients who also presented a PNP [10],[14],[46]. Nine additional index patients of our cohort showed evidence for a polyneuropathy, but no mutation was found in these cases. Thus, there probably are other genes which cause a combined phenotype of MFM and polyneuropathy.

The most common cardiac symptoms in our cohort were arrhythmias (83%), followed by conduction defects (50%). This is in accordance with a 10-year longitudinal study in *DES* patients, in which conduction defects were the most common cardiac manifestation [59].

Taking the whole cohort into account, five patients complained about swallowing problems (one carried a *DES* p.Arg350Pro mutation).

The occurrence of multisystemic symptoms might be explained by the expression profiles of the proteins encoded by the MFM genes. However, the expression of proteins remains partially unknown, e.g. FLNC expression in the vestibulocochlear nerve.

### Muscle MRI findings compared to the literature

For detailed values of the different muscles compared to the literature see Table 3[51]. Patient F1.III.1 and patient F2.III.4 harbour the same mutation in *DES* (Table 1, Figure 1). Patient F2.III.4 showed an equal pattern of muscle involvement as described before even after 32 years of disease duration. However, the pattern seen in patient F1.III.1 is different, especially his gluteus minimus, quadriceps, and soleus muscle were far more involved than in previously reported cases. Patient F7.III.4 carries a *FLNC* mutation and showed a relatively typical pattern of muscle involvement in the MRI, only the severe involvement of the rectus femoris and semitendinosus and the spared tibialis anterior muscle was rather unusual. In the patient with a *MYOT* mutation (patient F10.1) we would have expected the involvement of the tibialis anterior muscle, because of the severely involved posterior compartment, as well as the vastus medialis and intermedius muscles which are described to be one of the most affected in the literature and are only mildly involved here. In the patient with a *ZASP* mutation (patient F.12.III.3) we found a rather mild involvement at muscle MRI, but as was described before biceps femoris and semimembranosus muscles were most severely affected.

## Conclusion

We conclude that multisystemic involvement frequently occurs in MFM. Most important are the cardiac and respiratory symptoms, but also polyneuropathies, hearing loss and bulbar symptoms can occur. *BAG3* should be included in the genetic workup of MFM patients, even in cases with an adult onset and a mild phenotype. In 29% of all biopsies no aggregations were found at the light microscopic level, however, typical ultrastructural findings were present, underlining the importance of electron microscopy in the diagnosis of MFM. In contrast, 76% of the patients/biopsies without a genetic identification showed distinct protein deposits in the cytoplasm of the muscle cells. This high rate and our percentage of identified mutations (37%) lead to the conclusion that more causative genes for MFM are still to be found. NGS might be helpful in achieving this aim.

## Competing interests

The authors declare that they have no conflicts of interest.

## Authors’ contributions

AS carried out the acquisition and interpretation of clinical, histopathological and imaging data, participated in the molecular genetic and histopathological studies and drafted the manuscript. SS was involved in collecting and analysing clinical and histopathological data and revised the manuscript. JEB carried out molecular genetic studies and drafted parts of the manuscript. CL participated in acquisition and interpretation of clinical and histopathological data and revised the manuscript. JB was involved in collecting and analysing clinical data and revised the manuscript. RAK was involved in collecting and analysing clinical data and revised the manuscript. AF participated in collecting and analysing clinical and imaging data and revised the manuscript. RA was involved in collecting and analysing clinical data and revised the manuscript. PVdB was involved in collecting and analysing clinical and histopathological data and revised the manuscript. JJM participated in the acquisition and interpretation of clinical and histopathological data and revised the manuscript. PDJ was involved in collecting clinical data and revised the manuscript. ENJ participated in collecting and analysing of histopathological data and revised the manuscript. OM participated in the acquisition and interpretation of clinical data and revised the manuscript. MD participated in collecting and analysing of clinical and imaging data and revised the manuscript. MB participated in collecting and interpretation of clinical data and revised the manuscript. JMS participated in the acquisition and interpretation of histopathological data and revised the manuscript. MV participated in collecting and analysing of clinical and histopathological data and revised the manuscript. JBS participated in the acquisition and interpretation of clinical data and revised the manuscript. JW participated in the acquisition and interpretation of histopathological data and revised the manuscript. WK carried out the molecular genetic studies and drafted and revised the manuscript. KGC conceived, coordinated and supervised the study, participated in the collection and analysing of the clinical, histopathological, imaging and genetic data, and corrected the manuscript. All authors read and approved the final manuscript.

## Additional files

## Supplementary Material

Additional file 1: Non-pathogenic or unclassified variants identified by means of NGS panel diagnostics in the MFM-causing genes. Click here for file

Additional file 2: Review of multisystemic symptoms described in literature. Click here for file
