# Supplementary material for: Unusual multisystemic involvement and a novel BAG3 mutation revealed by NGS screening in a large cohort of myofibrillar myopathies
Source: Orphanet J Rare Dis. 2014 Aug 1;9:121. doi: 10.1186/s13023-014-0121-9 (PMC4347565; doi:10.1186/s13023-014-0121-9)
Supplement: Additional file 1: — Non-pathogenic or unclassified variants identified by means of NGS panel diagnostics in the MFM-causing genes. [file s13023-014-0121-9-S1.pdf]

Additional file 1: Non-pathogenic or unclassified variants identified by means of NGS panel diagnostics in the MFM-causing genes.

Pat.	Gene	Exon	Variant	SNP database	Predicted as	Conservation status	NHLB1	1000GP
F13.1	*ZASP*	4	c.664G>A, p.Ala222Thr*	rs139922045, MAF <0.1 %	predicted inconsistently	AA highly, Nt weakly	4/8596 (European American population)	not found
F15.1	*FLNC*	21	c. 3721C>T, p.Arg1241Cys	rs146953558, MAF 0,95%	benign	AA highly, Nt weakly	81/8433 (European American population)	5/753 (European population)
F17.1	*FLNC*	23	c.4022G>A, p.Arg1341Gln	rs149641783, MAF 0,18%	predicted inconsistently	AA highly	15/8425 (European American population)	2/756 (European population)
F19.1	*FLNC*	34	c.5578C>T, p.Arg1860Cys	rs181067717, MAF 0,62%	probably/possibly damaging	AA highly, Nt moderately	52/8320 (European American population)	3/755 (European population)
F20.1	*FLNC*	40	c.6595G>A, p.Gly2199Arg	rs368977589, MAF 0,012%	predicted inconsistently	AA and Nt highly	1/8357 (European American population)	not found
F23.1	*TTN*	343	c.95297C>T, p.Ser31766Phe	rs191484894, MAF 0.2 %	disease causing	AA and Nt highly	16/8334 (European American population)	2/756 (European population)

Predicted as, here the results of the prediction programs PolyPhen-2 and Mutation Taster are shown; Conservation status, conservation of the changed amino acid (AA) and nucleotide (Nt) using data of Alamut; NHLB1, frequency of the variant according to the database of NHLBI exome sequencing project (ESP); 1000GP, frequency of the variant according to the database of the 1000 genome project.*, the disease did not segregate in the family; MAF, minor allele frequency; predicted inconsistently, the mutation is predicted to be disease causing by one programme and predicted to be benign by the other; -, not performed; for abbreviations of genes see text.
