# Supplementary material for: Unusual multisystemic involvement and a novel BAG3 mutation revealed by NGS screening in a large cohort of myofibrillar myopathies
Source: Orphanet J Rare Dis. 2014 Aug 1;9:121. doi: 10.1186/s13023-014-0121-9 (PMC4347565; doi:10.1186/s13023-014-0121-9)
Supplement: Additional file 2: — Review of multisystemic symptoms described in literature. [file s13023-014-0121-9-S2.pdf]

Additional file 2.pdf – Review of multisystemic symptoms described in literature.

**Gene/ Symptom**	***DES***	***CRYAB***	***FLNC***	***MYOT***	***ZASP***	***FHL-1***	***BAG3***	***DNAJB6***	***TTN***
**Cardiac involvement**	**AF:**<br>Arias et al. 2006<br>**Arrhythmias:**<br>[34, 41]<br>**Conduction defects:**<br>[8, 27, 37, 39, 40]<br>Cao et al. 2012<br>**MI/TI:**<br>Dalakas et al. 2000<br>**HCM:**<br>[33]<br>**DCM:**<br>[20]<br>**RCM:**<br>[32]<br>Vrabie et al. 2005<br>Arbustini et al. 2006<br>**ARVC:**<br>[38]<br>**HTx:**<br>Klauke et al. 2010<br>**SCD:**<br>[22, 29, 35]	[9, 31, 47, 61]<br>Iganaki et al. 2006<br>van der Smagt et al.2014	[30, 42, 60]<br>Tasca et al. 2012<br>Avila-Smirnow et al. 2010	[4, 10]	[12, 49]<br>Vatta et al. 2003	Binder et al. 2012	[14, 36, 46, 48, 58]	[15]	[26]
**Respiratory involvement**	[8, 20, 27, 32, 33, 34, 35, 37, 39, 40, 44]<br>Dagvadorj et al. 2003<br>Dalakas et al. 2000	[19, 28, 31]<br>Del Bigio et al. 2011	[11, 30, 60]<br>Tasca et al. 2012	[4]		Feldkirchner et al. 2013	[14, 36, 46, 48, 58]		[16, 26, 45]<br>Edström et al. 1990
**Poly-neuropathy**			[11]	[10]	[12]	[13]	[14, 36, 46, 48]		
**Dysphagia**	[8, 20, 22, 27, 29, 33, 37, 39, 40,43, 44]<br>Dagvadorj et al. 2003	[9, 28, 31, 47]							[45]
**Dysarthria**	[22, 27, 33]	[31]	Tasca et al. 2012	[4]			[14]		[45]
**Cataract**	[22, 33]	[9, 31]<br>van der Smagt et al. 2014							
**Diarrhoe**	[37, 39, 40, 41]		[42, 60]						
**Scleroderma**		[28]							
**Hearing impairment**	[39, 40]				[49]				

**Gynaeko-mastia**	[29, 37]	[47]							
**Lipo-dystrophy**						Feldkirchner et al. 2013			
**Cerebellar ataxia**			Tasca et al. 2012						

AF, atrial fibrillation; TI/MI, tricuspid or mitral insufficiency; HCM, hypertrophic cardiomyopathy; DCM, dilated cardiomyopathy; RCM, restrictive cardiomyopathy; ARVC, arrhytmogenic right ventricular cardiomyopathy; HTx, heart transplantation; SCD, sudden cardiac death, for abbreviations of genes see text.

References:

Arias M, Pardo J, Blanco-Arias P, Sobrido M, Arias S, Dapena D, Carracedo A, Goldfarb LG, Navarro C: **Distinct phenotypic features and gender-specific diseasemanifestations in a Spanish family with desmin L370P mutation.** *Neuromuscul Disord* 2006, **16:**498-503.

Cao L, Hong D, Zhu M, Li X, Wan H, Hong K: **A novel heterozygous deletion-insertion mutation in the desmin gene causes complete atrioventricular block and mild myopathy.** *Clin Neuropathol* 2013, **32:**9-15.

Dalakas MC, Park K, Semino-Mora C, Lee HS, Sivakumar K, Goldfarb LG: **Desmin myopathy, a skeletal myopathy with cardiomyopathy caused by mutations in the desmin gene.** *N Engl J Med* 2000, **342:**770-780.

Vrabie A, Goldfarb LG, Shatunov A, Nagele A, Fritz P, Kaczmarek I, Goebel HH: **The enlarging spectrum of desminopathies: new morphological findings, eastward geographic spread, novel exon 3 desmin mutation.** *Acta Neuropathol* 2005, **109:**411-417.

Arbustini E, Pasotti M, Pilotto A, Pellegrini C, Grasso M, Previtatli S, Repetto A, Bellini O, Azan G, Scaffino M, Campana C, Piccolo G, Vigano M, Tavazzi L: **Desmin accumulation restrictive cardiomyopathy and atrioventricular block associated with desmin gene defects.** *Eur J Heart Fail* 2006, **8:**477-483.

Klauke B, Kossmann S, Gaertner A, Brand K, Stork I, Brodehl A, Dieding M, Walhorn V, Anselmetti D, Gerdes D, Bohms B, Schulz U, zu Knyphausen E, Vorgerd M, Gummert J, Milting H: **De novo desmin-mutation N116S is associated with arrhythmogenic right ventricular cardiomyopathy.** *Hum Mol Genet* 2010, **19:**4595–4607.

Dagvadorj A, Goudeau B, Hilton-Jones D, Blancato JK, Shatunov A, Simon-Casteras M, Squier W, Nagle JW, Goldfarb LG, Vicart P: **Respiratory insufficiency in desminopathy patients caused by introduction of proline residues in desmin c-terminal α-helical segment.** *Muscle Nerve* 2003, **27:** 669–675.

Dagvadorj A, Olivé M, Urtizberea JA, Halle M, Shatunov A, Bönnemann C, Park KY, Goebel HH, Ferrer I, Vicart P, Dalakas MC, Goldfarb LG: **A series of West European patients with severe cardiac and skeletal myopathy associated with a de novo R406W mutation in desmin.** *J Neurol* 2004, **251:**143–149.

Iganaki N, Hayashi T, Arimura T, Koga Y, Takahashi M, Shibata H, Teraoka K, Chikamori T, Yamashina A, Kimura A: **Alpha B-crystallin mutation in dilated cardiomyopathy.** *Biochem Biophys Res Commun* 2006, **342:**379–386.

van der Smagt JJ, Vink A, Kirkels JH, Nelen M, ter Heide H, Molenschot MMC, Weger RA, Schellekens PAW, Hoogendijk J, Dooijes D: **Congenital posterior pole cataract and adult onset dilating cardiomyopathy. Expanding the phenotype of αB-crystallinopathies.** *Clin Genet* 2014, **85:**381-385.

Del Bigio MR, Chudley AE, Sarnat HB, Campbell C, Goobie S, Chodirker BN, Selcen D: **Infantile Muscular Dystrophy in Canadian Aboriginals is an αBCrystallinopathy.** *Ann Neurol* 2011, **69:**866–871.

Tasca G, Odgerel Z, Monforte M, Aurino S, Clarke NF, Waddell LB, Udd B, Ricci E, Goldfarb LG: **Novel FLNC mutation in a patient with myofibrillar myopathy in combination with late-onset cerebellar ataxia.** *Muscle Nerve* 2012, **46:**275–282.

Avila-Smirnow D, Béhin A, Gueneau L, Claeys K, Beuvin M, Goudeau B, Richard P, Yaou RB, Romero NB, Mathis S, Voit T, Eymard B, Gil R, Fardeau M, Bonne G: **A novel missense FLNC mutation causes arrhythmia and late onset myofibrillar myopathy with particular histopathology features [abstract].** *Neuromuscul Disord* 2010, **20:**596–680.

Vatta M, Mohapatra B, Jimenez S, Sanchez X, Faulkner G, Perles Z, Sinagra G, Lin JH, Vu TM, Zhou Q, Bowles KR, Di Lenarda A, Schimmenti L, Fox M, Chrisco MA, Murphy RT, McKenna W, Elliott P, Bowles NE, Chen J, Valle G, Towbin JA: **Mutations in *Cypher/ZASP* in Patients with dilated cardiomyopathy and left ventricular non-compaction.** *J Am Coll Cardiol* 2003, **42:** 2014–2027.

Binder JS, Weidemann F, Schoser B, Niemann M, Machann W, Beer M, Plank G, Schmidt A, Bisping E, Poparic I, Lafer I, Stojakovic, Quasthoff S, Vincent JB, Rienmueller R, Speicher MR, Berghold A, Pieske B, Windpassinger C: **Spongious hypertrophic Cardiomyopathy in patients with mutations in the Four-and-a-Half LIM Domain 1 Gene.** *Circ Cardiovasc Genet* 2012, **5:**490-502.

Feldkirchner S, Walter MC, Müller S, Kubny C, Krause S, Kress W, Hanisch FG, Schoser B, Schessl J: **Proteomic characterization of aggregate components in an intrafamilial variable FHL1-associated myopathy.** *Neuromuscul Disord* 2013, **23:**418–426.

Edstrom L, Thornell LE, Albo J, Landin S, Samuelsson M: **Myopathy with respiratory failure and typical myofibrillar lesions.** *J Neurol Sci* 1990, **96:**211-228.
